# Usability assessment of drug-linking laboratory data listed on prescriptions for outpatients of chiba university hospital

**DOI:** 10.1038/s41598-021-81344-y

**Published:** 2021-01-18

**Authors:** Iichiro Yokoyama, Hiroki Yamaguchi, Kaori Yamazaki, Misato Sekiya, Sayaka Arai, Takako Nakamura, Takaaki Suzuki, Itsuko Ishii

**Affiliations:** Division of Pharmacy, Chiba University Hospital, 1-8-1 Inohana, Chuo-ku Chiba-shi, Chiba, 260-8677 Japan

**Keywords:** Health care, Medical research

## Abstract

To evaluate the impact of pharmacotherapy on efficacy and safety by providing laboratory data information linked to medicines on outpatient prescriptions from the hospital to the community pharmacy. Beginning on October 28, 2014, standardized laboratory data and drug-linking laboratory data were included with outpatient prescriptions at our hospital. We have created a database of drug-linking laboratory data for all drugs that can be prescribed in Japan. We counted the number of prescription inquiries related to laboratory data from community pharmacies, including those leading to prescription changes. Before laboratory data were listed on outpatient prescriptions, 4 prescription inquiries from community pharmacies per year were related to laboratory data. After our hospital started to list laboratory data, this number rose to 643, 576, 563, and 847 in the first, second, third, and fourth year (*P* < .05). Of these, 132, 143, 152, and 224 inquiries resulted in prescription changes. Listing laboratory data on outpatient prescriptions avoided 153 contraindications and 84 exacerbations of adverse drug reactions in four years by a prescription inquiry that had never been done before. The efficacy and safety of pharmacotherapy can be improved by listing relevant laboratory data on outpatient prescriptions.

## Introduction

The efficacy and safety of pharmacotherapy is maintained by a two-step check during the medication cycle. Phase 1 is when the physician prescribes, and phase 2 is when the pharmacist checks the validity of the prescription (prescription check). However, prescribing errors and adverse drug reactions (ADRs) are serious hazards for patients all over the world. US reports estimate that there are at least 1.5 million preventable ADRs per year in the United States^1^. Most prescribing errors, and a majority of ADRs, occur during the prescription phase of the medication cycle^2^. In particular, much inappropriate prescribing is related to laboratory data^3^. For example, regarding exacerbation of ADRs, potassium supplementation was prescribed for a patient who was hyperkalemic^4^. An overdose would result from failure to adjust the dose of gentamicin in a patient with impaired renal function; 70% of prescriptions containing renally excreted drugs had inappropriately high doses^5^. Other reports include inappropriate overdoses^6^ and failure to perform necessary clinical examinations associated with the drugs^7,8^.

Laboratory data are important for drug selection and ADR monitoring, but usually drug and laboratory data are not linked^9,10^. If the drug and laboratory data systems work together, this will prevent many prescribing errors^11,12^. There are few such reports^13–16^, but they are not comprehensive. Linking drugs and laboratory data may improve the quality of both pharmacotherapy and clinical investigation, but most hospitals do not have such linkage systems^17–19^. This is because there is no standardized or innovative drug and laboratory data link rule, and maintenance is difficult because many new drugs are released to the market every year^17^.

The support of information technology is indispensable to improve the safety of pharmacotherapy^1^. There are ways to alert the physician to potential problems when prescribing, but a blizzard of false-positive alerts and alerts that are not tailored to the patient have plagued physicians^20^. The availability of many unsophisticated alerts has led to important alerts being missed^21,22^. Therefore, it is important to improve the quality of the prescription check performed by a pharmacist to enable a more comprehensive check.

The information that physicians are required to provide when prescribing medication in Japan is the patient's name, age, name of the medication, dosage, duration of administration, date of prescription, expiration date, name and location of the medical institution, and the physician's signature. The information that must be included on the prescription is the same for outpatients and inpatients. The physician hands the prescription to the outpatient. Community pharmacists perform prescription checks on prescriptions brought by outpatients to a community pharmacy. If a prescription is inappropriate, the pharmacist then queries the prescriber to improve/correct it^23,24^. Community pharmacists are able obtain much less patient information than hospital pharmacists. A recent systematic review revealed that there are no reports of interventions using renal function data at community pharmacies^25^. Therefore, it is difficult for community pharmacists to perform high-quality prescription checks tailored to the individual patient. Although the use of laboratory data is considered useful during prescription checks, there are no reports on laboratory data provided by the hospital to the community pharmacy or on prescription check linking drugs and laboratory data^26^.

This study sought to verify whether providing laboratory data information linked to drugs from the hospital to the outpatient community pharmacy could enable community pharmacists to use laboratory data for prescription checks and to ultimately avoid ADRs and contraindications.

## Methods

### Laboratory data listing on outpatient prescriptions

**Figure 1:**
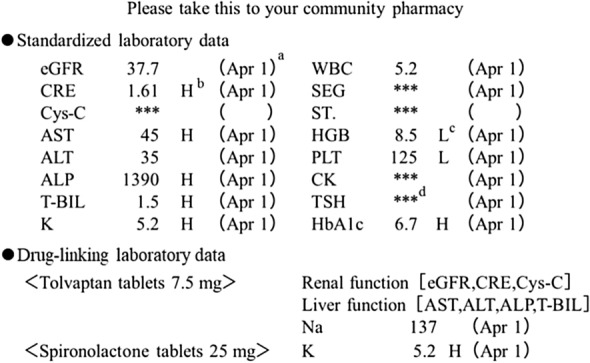
Example of laboratory data on an outpatient prescription. The laboratory data listed on the prescription are the latest data available at the time of prescription order registration. When the prescribing physician registers the prescription order, the laboratory data information is printed on a separate sheet at the same time as when the prescription is printed from the electronic medical records system. Both items are given to the patient by the prescribing physician. The patient then brings the laboratory data information and the prescription to the community pharmacy and submits them together. The symbols in the figure are as follows: (**a**) (Apr 1) indicates the measurement date. (**b**) H indicates a value higher than the reference range. (**c**) L indicates a value lower than the reference range. (**d**) *** indicates that there is no measured value within the last 100 days. Laboratory items are abbreviated as follows: eGFR, estimated glomerular filtration rate (mL/min); CRE, creatinine (mg/dL); Cys-C, cystatin C (mg/dL); AST, aspartate aminotransferase (U/L); ALT, alanine aminotransferase (U/L); ALP, alkaline phosphatase (U/L); T-BIL, total bilirubin (mg/dL); K, potassium (mmol/L); WBC, white blood cell count (× 1000/µL); SEG, segmented cell (%); ST., stab cell (%); HGB, hemoglobin (g/dL); PLT, platelet count (× 1000/µL); CK, creatine kinase (U/L); TSH, thyroid-stimulating hormone (µIU/mL); HbA1c, hemoglobin A1c (%); Na, sodium (mmol/L).

Our hospital began listing laboratory data on outpatient prescriptions on October 28, 2014. Both standardized and drug-linking laboratory data were listed on every prescription (Fig. 1). A standardized laboratory data is a listing method of a laboratory data that is commonly listed on all prescriptions, regardless of the prescribed medication. The drug-linking laboratory data is a listing method in which the laboratory data that require special attention for each prescribed medication are displayed with the name of the medication. Renal function, liver function, and myelosuppression, which are associated with multiple standardized laboratory data, were displayed as "Renal function[eGFR,CRE,Cys-C]" in the drug-linking laboratory data, and the values were confirmed by the standardized laboratory data. These are an attempt to reduce the display area.

### Selection criteria for standardized laboratory data

Selection criteria for the standardized laboratory data (the laboratory items commonly listed on all prescriptions) were based on manuals for serious ADRs issued by the Japanese Ministry of Health, Labour and Welfare (https://www.mhlw.go.jp/stf/seisakunitsuite/bunya/kenkou_iryou/iyakuhin/topics/tp061122-1.html, 10/1/2018). This manual comprehensively summarizes the treatment and discrimination methods utilized by physicians and pharmacists for severe ADRs. The 16 laboratory data items (Fig. 1) were chosen to enable early detection of ADRs for which early detection from subjective symptoms is difficult.

### Selection criteria for drug-linking laboratory data

Drug-linking laboratory data were selected if they met any of the following 3 criteria: (1) Laboratory data were specifically described in the “Warnings,” “Contraindications,” and “Relative Contraindications” section of a drug’s package insert, meaning summary of product characteristics. (2) Laboratory data could be used to identify diseases and symptoms described in the “Warnings,” “Contraindications,” and “Relative Contraindications” section of the drug’s package insert. (3) Dosage for a drug was dependent on renal function: either the “Precautions Related to Dosage and Administration” section of a drug’s package insert specified dosage according to renal function, or the drug was listed in the Appendix to the Guideline for Medical Care and Treatment of Drug-Induced Nephropathy 2016 (https://cdn.jsn.or.jp/academicinfo/report/CKD-guideline2016.pdf, 4/1/2020).

When selected according to these criteria, 460 drugs and 599 laboratory items were among 1698 drugs that could be prescribed for outpatients in our hospital. The laboratory items included the following 20 items: Renal function, liver function, myelosuppression, T-BIL, amylase, white blood cells (WBC), neutrophils, erythrocytes, hemoglobin, platelet count, lymphocytes, serum potassium, serum sodium, serum magnesium, serum calcium, serum albumin, triglycerides, cholinesterase, glucose, and prothrombin time-international normalized ratio.

### Creation of database for drug-linking laboratory data

We started to target contraindications and warnings on the drug label. We picked up about 50,000 contraindications and warnings on the package insert of about 20,000 prescription drugs approved in Japan, and analyzed the contraindications and warnings related to laboratory data. The selection criteria for drug-linking laboratory data described above were created from the analysis results. We have built into the program the rules that we were able to get from about 50,000 contraindications and warnings. The database items are the drug standard code, drug name, laboratory item code, laboratory item name, and the reason for the link (contraindication or warning). When the serum calcium value was registered, albumin for calculating the corrected calcium value was also registered. From the completed program, extraction work was carried out for new drugs and package inserts revised every month, and a system for obtaining extraction results as "database for drug-linking laboratory data" was completed. The monthly maintenance work method of the database for drug-linking laboratory data obtained was to mechanically extract the difference from the previous month and visually check whether there was any problem in the mechanical extraction result. All the drugs that can be prescribed in Japan can be updated. As of May 10, 2020, 5887 laboratory data items have been registered for 3808 drugs.

### Analysis of laboratory-data-related prescription inquiries leading to prescription changes

All prescription inquiries from community pharmacies were received by the hospital pharmacist over the phone, who consulted with a medical doctor if necessary, and then responded to the inquiry. One outpatient prescription includes one or more medicinal product for one patient. One prescription inquiry is one medicinal product inquiry for one patient. All prescription inquiries were recorded in Excel files. Classification and evaluation of prescription inquiries were performed by one pharmacist and then double-checked by another pharmacist. For all prescription inquiries that led to a prescription change, the following were analyzed: content of the prescription inquiry, laboratory items used, laboratory data listing method (standardized or drug-linking) that triggered the inquiry, and the reason for the prescription inquiry. We judged the reason to be an ADR in the following cases: first, when the abnormal laboratory data or symptom considered to be caused by the drug was consistent with known ADR information and resolved after appropriate intervention for the suspected drug ADR; second, when a prescription was changed on suspicion of ADR in consultation with a medical doctor.

### Statistical processing

Category variables between the two groups were compared using the Fisher exact test. If the significance level was less than 5% (*P* < 0.05), the difference was judged to be significant. All statistical analyses were performed using IBM SPSS Statistics Ver. 22 (IBM Japan Co., Ltd., Tokyo, Japan).

## Results

### Content of prescription inquiries at baseline

**Figure 2:**
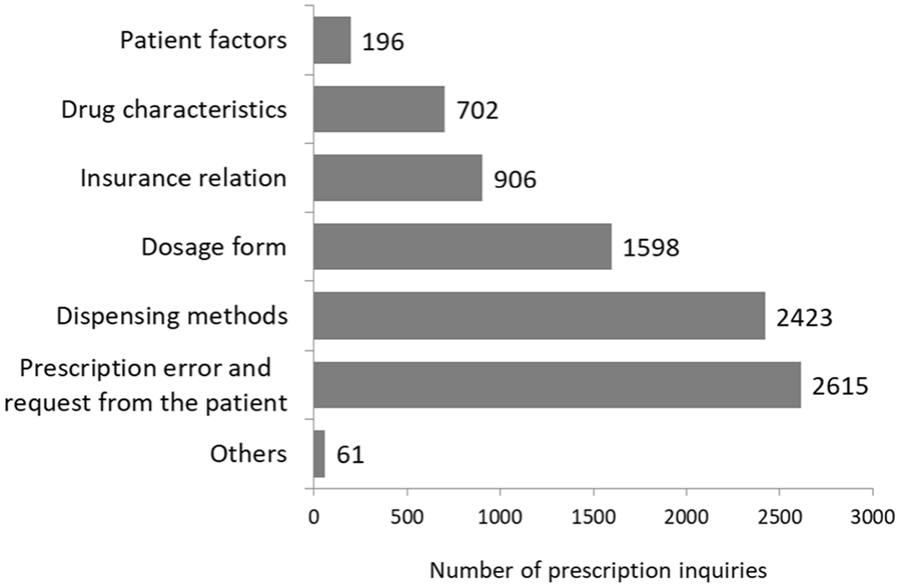
Prescription Inquiries Prior to Listing Laboratory Data on Prescriptions. We categorized prescription inquiries from community pharmacies at baseline (November 2013 through October 2014), before the hospital began to list laboratory data on prescriptions. Patient factors include age, sex, medication history, and past medical history not including laboratory data. Drug characteristics include dose timing and starting dose. Insurance-related inquiries are those concerning payment.

From November 2013 through October 27, 2014, a total of 239,471 outpatient prescriptions were written at our hospital and 8501 prescription inquiries (3.6%) were received from community pharmacies. When the prescription inquiries were classified by content (Fig. 2), prescribing errors and requests from patients accounted for the largest number (2615 cases, 30.8%). Patient-related factors such as age, sex, and medication history accounted for 196 prescription inquiries (2.3%). However, no inquiry during this period used patient laboratory data.

### Prescription changes resulting from prescription inquiries due to laboratory data

Before laboratory data were listed on outpatient prescriptions (11/2013–10/27/2014), only 4 prescription inquiries from community pharmacies were on issues related to laboratory data. This was because the community pharmacist found out that the patients were on dialysis, and therefore has decreased renal function. After our hospital started to list standardized laboratory data and drug-linking laboratory data on prescriptions, this number rose to 643, 576, 563, and 847 in the first, second, third, and fourth year. Of these, 132, 143, 152, and 224 inquiries resulted in prescription changes (Table 1). Both the number of prescription inquiries and the number of prescription changes increased significantly as a result of listing laboratory data on prescriptions. Table 2 shows some representative examples of prescription inquiries that led to prescription changes.

**Table 1:** Number of prescription inquiries and prescription changes related to laboratory data.

	Baseline (11/2013–10/27/2014)	Year 1 (11/2014–10/2015)	Year 2 (11/2015–10/2016)	Year 3 (11/2016–10/2017)	Year 4 (11/2017–10/2018)	Totals, Year 1–Year 4
Total prescriptions	239 471	245 638	248 440	251 896	260 448	1 006 422
Prescription inquiries	4	643 (*P* < .05)	576 (*P* < .05)	563 (*P* < .05)	847 (*P* < .05)	2629
Inquiries per 1000 prescriptions	0.017	2.62	2.32	2.24	3.25	2.61
Prescription changes	2	132 (*P* < .05)	143 (*P* < .05)	152 (*P* < .05)	224 (*P* < .05)	651
Changes per 1000 prescriptions	0.0084	0.54	0.58	0.60	0.86	0.65

During baseline from 11/2013 to 10/27/2014, neither standardized laboratory data nor drug-linking laboratory data was listed on prescription. Year 1, 2, 3 and 4, both type of laboratory data (standardized and drug-linking) were listed on prescription.

A statistically significant difference was observed between baseline and each year (*P* < .05, Fisher's exact test).

**Table 2:** Examples of prescription changes due to prescription inquiries related to laboratory data.

Nature of prescription inquiry	Drug	Laboratory data	Prescription inquiry	Result
To avoid exacerbation of adverse drug reactions	Glycyrrhizinate	Potassium 2.3 mmol/L	Serum potassium level decreased from 3.1 mmol/L to 2.3 mmol/L. Blood pressure is elevated. Pseudoaldosteronism is suspected, suggesting need to stop glycyrrhizinate or add an aldosterone antagonist	Medication added
	Eplerenone	Potassium 5.3 mmol/L	Since serum potassium level increased from 4.8 mmol/L to 5.3 mmol/L, eplerenone dose should be lowered	Dose lowered
	Diclofenac	CCr 9.7 mL/min	Diclofenac is contraindicated in severe renal dysfunction. Change to acetaminophen or tramadol is recommended. Prescribing physician decided to follow up without prescription change. However, creatinine clearance is impaired at next visit. Drug change is recommended again	Medication changed
	Voriconazole	Alkaline phosphatase 5368 U/L	Alkaline phosphatase value 2 weeks earlier is high and is still increasing. Liver dysfunction caused by voriconazole is suspected, but there is no blood test data for that day	Dose lowered
	Distigmine	Cholinesterase 154 U/L	Cholinesterase level is decreasing, the medication should be stopped	Medication stopped
To avoid adverse drug reactions	Dabigatran	CCr 25.9 mL/min	Contraindicated in severe renal dysfunction. Proposal for change to apixaban	Medication changed
	Olanzapine	HbA1c 9.1%	Contraindicated in diabetes mellitus with original prescription	Medication changed
	Sitagliptin	CCr 25.1 mL/min	Proposal to start at 12.5 mg	Dose lowered
To improve the effect of drug therapy	Amoxicillin	Neutrophil 252/µL	Since antimicrobial drugs that cover *Pseudomonas aeruginosa* are required for treatment of febrile neutropenia, we suggested a change to levofloxacin	Medication changed

### Analysis of laboratory-data-related prescription inquiries leading to prescription changes

The subset of prescription inquiries that led to prescription changes was analyzed from the following viewpoint. ADRs accounted for more than 95% of the prescription inquiries, and approximately 13% of the resulting changes prevented exacerbations of ADRs through early detection. Over the 4-year study period, a total of 84 exacerbations of ADRs were avoided (Table 3a).

**Table 3:** Analysis of prescription changes due to prescription inquiries related to laboratory data.

Classification	Baseline (11/2013–10/27/2014)	Year 1 (11/2014–10/2015)	Year 2 (11/2015–10/2016)	Year 3 (11/2016–10/2017)	Year 4 (11/2017–10/2018)
(a) *Nature of prescription inquiries*					
To avoid adverse drug reactions	2	110	117	128	189
To avoid exacerbation of adverse drug reactions	0	19	24	17	24
To improve the effect of drug therapy	0	3	2	7	11
Total	2	132	143	152	224

Laboratory items	Baseline (11/2013–10/27/2014)	Year 1 (11/2014–10/2015)	Year 2 (11/2015–10/2016)	Year 3 (11/2016–10/2017)	Year 4 (11/2017–10/2018)
(b) *Laboratory items that triggered prescription inquiries*					
Renal function	2	108	116	131	196
Potassium	0	11	14	13	19
Liver function	0	4	1	1	3
Neutrophil count	0	2	0	0	2
White blood cell count	0	1	2	1	1
Hemoglobin	0	1	1	1	0
Sodium	0	1	1	0	0
Cholinesterase	0	1	0	1	0
Creatine kinase	0	1	0	1	0
Hemoglobin A1c	0	1	0	0	1
PT-INR	0	1	0	2	1
Calcium	0	0	7	1	1
Blood glucose level	0	0	1	0	0
Total	2	132	143	152	224

Listing method	Baseline (11/2013–10/27/2014)	Year 1 (11/2014–10/2015)	Year 2 (11/2015–10/2016)	Year 3 (11/2016–10/2017)	Year 4 (11/2017–10/2018)
(c) *Laboratory data listing method that triggered prescription inquiries*					
Standardized laboratory data	–	6	4	8	16
Drug-linking laboratory data	–	126	139	144	208
Total	–	132	143	152	224

Reason for listing	Baseline (11/2013–10/27/2014)	Year 1 (11/2014–10/2015)	Year 2 (11/2015–10/2016)	Year 3 (11/2016–10/2017)	Year 4 (11/2017–10/2018)
(d) *Selection criteria for drug-linking laboratory data*					
Dose adjustment according to renal function	–	90	99	115	158
Contraindication	–	36	40	28	49
Warning	–	0	0	1	1
Total	–	126	139	144	208

Over the 4-year study period, the laboratory item that triggered the most prescription changes (> 80%) was renal function, followed by serum potassium level (Table 3b). The drug-linking laboratory data listing system accounted for about 95% of prescription changes (Table 3c). Dose adjustment based on renal function accounted for more than 70% of prescription changes in all 4 years, and those due to contraindications accounted for 36 prescription changes in the first year, 40 in the second year, 28 in the third year, and 49 in the fourth year. Over the 4-year study period, a total of 153 contraindications were avoided (Table 3d).

## Discussion

In this study, we found that the quality of prescription checks at community pharmacies was improved by listing laboratory data on prescriptions for outpatients. Listing laboratory data on outpatient prescriptions improved drug efficacy and safety by avoiding 153 contraindications and 84 exacerbations of ADRs over a 4-year period, due to prescription inquiries that had not occurred in the past. In addition, our findings suggest that the drug-linking laboratory data listing method, which was introduced as an innovative link between drugs and laboratory data, proved useful for community pharmacists who are unfamiliar with the use of laboratory data.

At community pharmacies, it is important to hear from patients^27^, nevertheless, it can be difficult to obtain patient-related information or information about disease status. Therefore, the number of prescription inquiries based on patient-related factors is extremely small (Fig. 2). A feature of this study is that the community pharmacist has an established mechanism that can easily and quickly detect inappropriate prescribing. There are reports of systems for detecting prescriptions of otherwise contraindicated drugs because of potential drug interactions, age, and diseases^28^ and systems for checking laboratory data for specific drugs only^13–16^. However, there are no reports on a system that can comprehensively detect inappropriate prescribing related to laboratory data at a community pharmacy^29^, so we consider the system described in this study to be useful. And, this study is considered to have novelty different from the computerized physician order entry (CPOE) that has already been used. Although the alert system with linked drug and laboratory data is reported for a limited number of drugs^4–8^, this study created a link for all drugs, so it can cover reports of problems with various drugs and laboratory data. The reason why there is no database of sophisticated links covering all drugs is due to the lack of sophisticated linking rules^17–19^. In addition, considering that many drugs are introduced every year, it is considered that continuous maintenance is difficult^21^. In this study, the link rule is simplified so that it can be automatically extracted, and it is constructed so that monthly maintenance can be performed automatically. Therefore, it can respond to new drugs and revision of contraindications.

Analysis of the prescription inquiries that led to prescription changes suggested that 94.8% were related to the drug-linking laboratory data presented on the outpatient prescription (Table 3c), in turn indicating that drug-linking laboratory data may be the cornerstone of prescription inquiries. It is possible that the drug-linking laboratory data listing method may easily be used for prescription inquiries even by community pharmacists who are not accustomed to using laboratory data. The relevance of a link between the drug and the actual laboratory test data may have been clarified in this study.

Prolonged patient waiting time at the community pharmacy is already a problem^30^. However, this method, in which particularly important laboratory data are shown for each drug, is capable of minimizing the time required for a prescription check, so there is little concern of prolonged patient waiting time.

The rate of non-laboratory-data–related prescription inquiries from community pharmacies at our hospital was 3.6%, which is comparable to the rate of about 3% seen in Japan as a whole^31,32^. Consequently, we believe that drug efficacy and safety can be improved by introducing laboratory-data–related prescription inquiries to other medical institutions.

Renal function was the most likely type of laboratory data to trigger prescription inquiries (Table 3b). Because renal function declines with aging, many patients will require careful attention to renal function in terms of prescriptions. Moreover, about 20% of all drugs require dose adjustment according to renal function.

Pharmacist education is essential for improving the professional quality of their work^33^. Before our hospital started listing laboratory data on prescriptions for outpatients, we provided 4 educational opportunities for community pharmacists about prescription checks using this data, and we continue to provide educational opportunities. In addition, whenever a pharmacist in our hospital receives a prescription inquiry from a community pharmacy, we check with the community pharmacist about the laboratory data or investigation results and the presence or absence of symptoms associated with ADRs. The accumulation of these educational interventions is important for promoting the use of laboratory data in community pharmacies.

There are certain limitations of this study. First, even if a prescription inquiry was necessary, it is possible that the prescription inquiry was not sufficiently addressed. Second, for those prescription inquiries that did not result in prescription changes, we did not evaluate cases in which the prescribing physician gave verbal instructions to the patient via the community pharmacist and/or changed the prescription at the next visit. Third, this study did not include refill prescriptions, thus leaving out some vital information. In the future, if a patient’s latest laboratory data is at another clinic or hospital, the patient will be requested to bring it to the community pharmacy. This is expected to enable a prescription check equivalent to that in this study to be performed by introducing the drug-linking laboratory data database into the support system of the community pharmacy.

This is the first study to show that listing drug-linking laboratory data on outpatient prescriptions is useful for realizing individual optimization of pharmacotherapy in community pharmacies. However, there are major barriers to introducing the drug-linking laboratory data listing method to many medical institutions. Each institution needs to create its own database of drug-linking laboratory data, and updating the data takes a lot of time. Therefore, to make the methods used in this study widely available, we have created a database of drug-linking laboratory data for all prescription drugs in Japan. This database can automatically extract drugs and laboratory data based on contraindications and warnings in the package insert information, and can be updated for all drugs that can be prescribed in Japan, not just those adopted in the hospital. This database has made it possible to significantly reduce maintenance that requires a great deal of manpower. This database makes it easy to introduce drug-linking laboratory data at each medical institution. Introducing such a database will allow warnings to be issued at the time of prescribing, enable alerts on EHRs, and improve pharmaceutical care in the ward and hospital or community pharmacies. This will help ensure a reliable level of pharmacotherapy efficacy and safety.

## Conclusions

Efficacy and safety on pharmacotherapy can be improved by listing relevant laboratory data on outpatient prescriptions. We propose the use of drug-linking laboratory data databases to ensure a certain level of pharmacotherapy efficacy and safety in all pharmacies.

### Ethics approval

This study was conducted in compliance with the “Ethical Guidelines for Medical and Health Research Involving Human Subjects”. It was approved by the Ethics Review Committee at Chiba University School of Medicine by using the opt-out approach without the need for direct consent from each individual patients (Approval number: 3577).
